# Supplementary material for: Conserved and species-specific chromatin remodeling and regulatory dynamics during mouse and chicken limb bud development
Source: Nat Commun. 2021 Sep 28;12:5685. doi: 10.1038/s41467-021-25935-3 (PMC8479071; doi:10.1038/s41467-021-25935-3)
Supplement: Supplementary file 1 — Supplementary Information [file 41467_2021_25935_MOESM1_ESM.pdf]

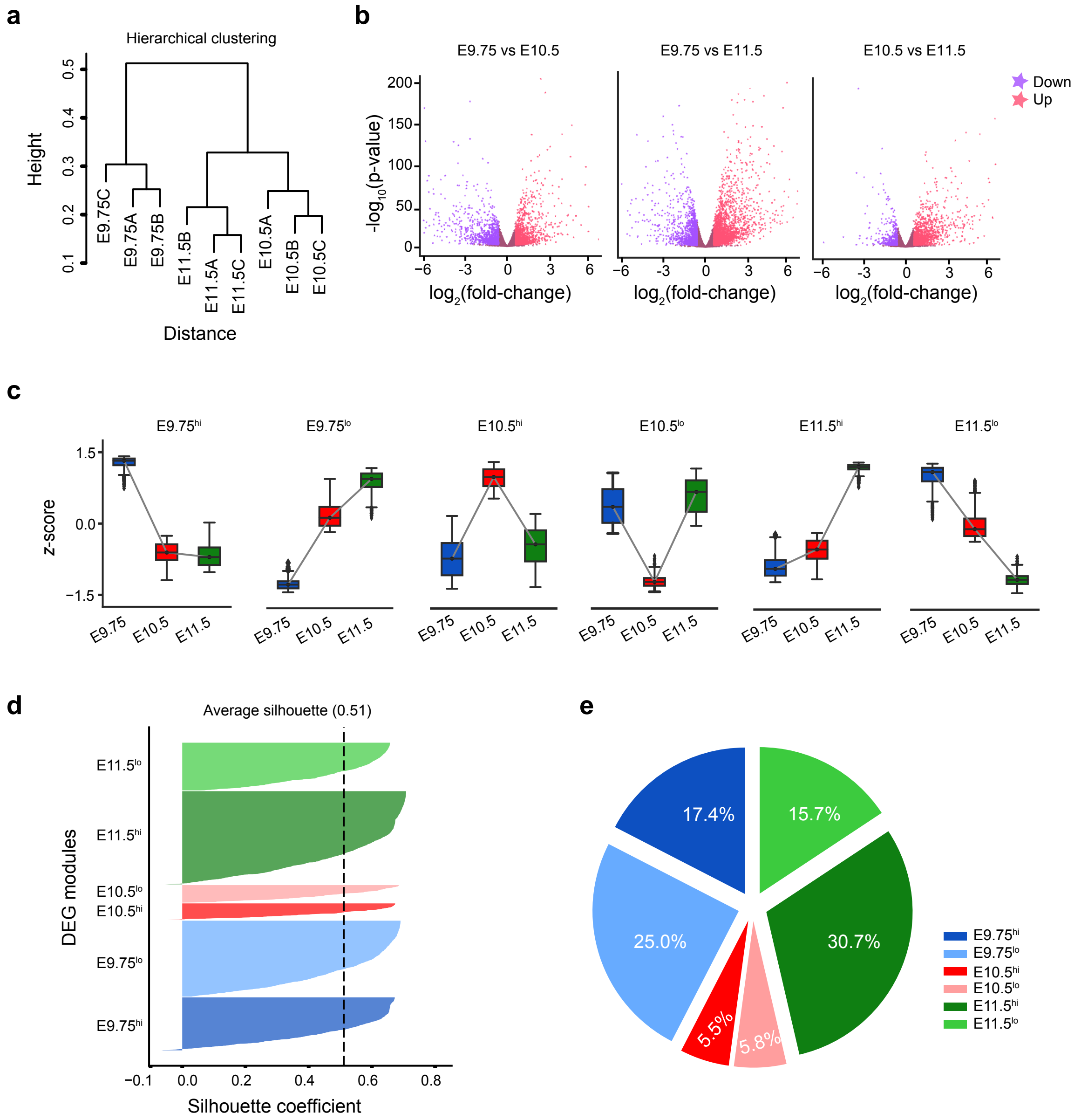


**Supplementary Figure 1. Analysis of stage-specific transcriptomes reveals the temporal dynamics of gene expression in developing mouse forelimb buds. a** Dendrogram represents the hierarchical clustering of samples. Replicates of the same stage (n=3) cluster together establishing low intra-stage variability. **b** Volcano plots showing statistical significance versus the magnitude of fold change for upregulated (right magenta) and downregulated genes (left purple) for the pairwise comparisons

indicated. **c** Box plots showing the quantification of relative expression of genes within the six DEG modules ($E9.75^{hi}$ n=755; $E9.75^{lo}$ n=1081; $E10.5^{hi}$ n=236; $E10.5^{hi}$ n=250, $E11.5^{hi}$ n=1327, $E11.5^{hi}$ n=680). The middle line represents the median ($50^{th}$ percentile), whereas the lower and upper hinges correspond to the first ($25^{th}$ percentile) and third ($75^{th}$ percentile) quartiles of the box plots, respectively. The upper whisker extends from the hinge to the largest value at 1.5 × inter-quartile range (IQR) of the hinge. Similarly, the lower whisker extends from the hinge to the smallest value within the 1.5x IQR. All other data points with values outside the whiskers are outliers and plotted individually. The minima and maxima of the box plots are determined after exclusion of outliers. **d** Plots show the distribution of the silhouette coefficients for the DEG modules. An average silhouette score of +0.51 highlights the high degree of inter-module separability. **e** Pie chart depicts the proportion of DEGs per module. DEG: differentially expressed genes. Source data are provided as a Source Data file.

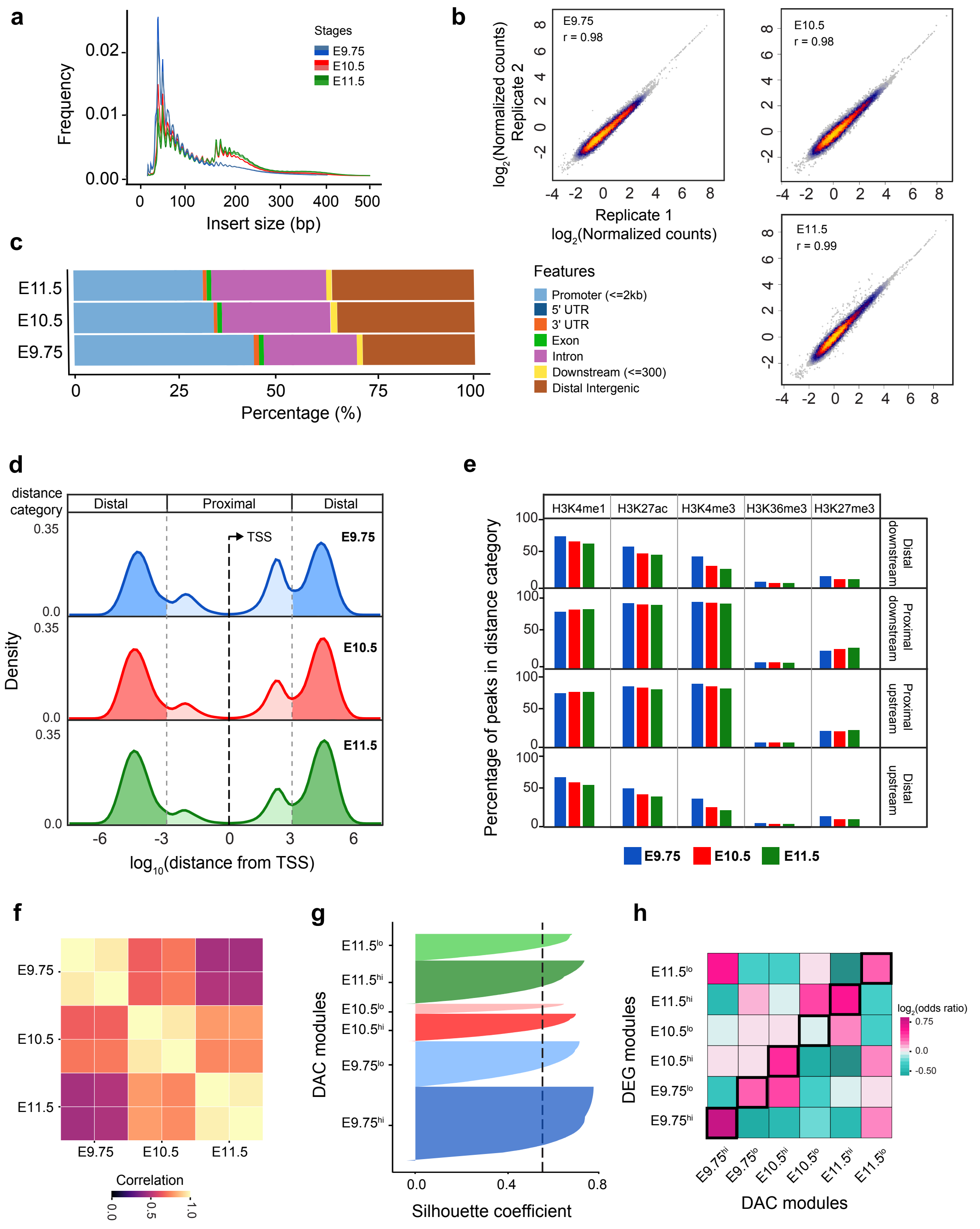


**Supplementary Figure 2. Temporal dynamics of the genome-wide chromatin accessibility during mouse forelimb bud development. a** Insert size frequency of ATAC-seq fragments shows the periodicity of insertions at the nucleosome boundaries

(~145bp interval). Colours indicate the different limb bud stages. **b** Scatter plots show the Pearson's correlation coefficient ($r \geq 0.97$) between replicates (n=2) for each mouse forelimb bud stage. The correlation reflects the linear relationship between the two replicates and was computed using log-transformed counts. Colours indicate the density of data points. **c** Distribution of accessible chromatin regions of individual stages in different genomic regions of mouse forelimb buds. Colours indicate the annotations to the different genomic regions. **d** Distances of accessible chromatin regions to the nearest TSS in mouse forelimb buds. Plotting using a $\log_{10}$ distance scale separates accessible chromatin regions into four distinct populations (proximal-downstream, proximal-upstream, distal-downstream, and distal-upstream). The distribution of accessible chromatin regions among the four categories remains very similar during forelimb bud development. **e** Bars represent the percentage overlap of accessible chromatin regions in each of the four categories with different histone modifications in mouse limb buds (from ENCODE). A very high overlap with at least one of the histone marks indicative of active enhancers and promoters (H3K27ac, H3K4me3, H3K4me1) is observed for all stages. **f** Spearman's correlation coefficient ($\rho$) for the pairwise combinations between replicates of each stage based on normalized accessibility scores for all DACs per sample. The correlation values range from 0 to 1 (scale bar). The replicates (n=2) per stage are highly correlated (>0.9) corroborating reproducibility. **g** Plot shows the silhouette coefficient for the six DAC modules. An average silhouette score of +0.56 highlights that the samples are far from the decision boundary between neighbouring modules. **h** Log-odds ratios for two-tailed Fisher's exact test of enrichment between pairs of DAC and DEG modules. These correspond to the p-values shown in Fig. 2i, where an adjusted p-value <0.05

is considered significant. DAC: differentially accessible chromatin regions, DEG: differentially expressed genes. Source data are provided as a Source Data file.

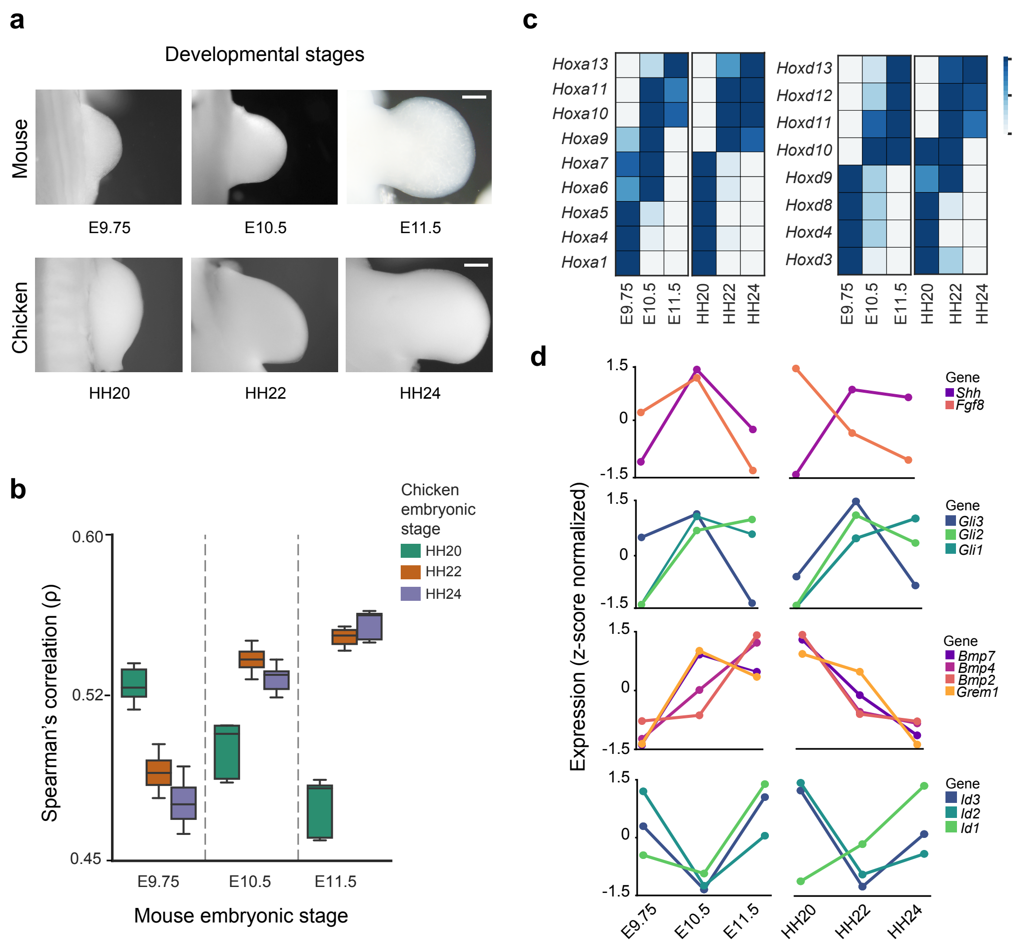


**Supplementary Figure 3. Developmental correspondence of mouse forelimb and chick wing bud stages. a** Correspondence was assessed by comparing mouse and chicken limb buds at E9.75/HH20, E10.5/HH22, and E11.5/late-HH24 (scale bars: 250$\mu$m). **b** Pairwise Spearman's correlation coefficient (ρ) computed using the expression profiles of highly variable orthologues genes (n=1226) in mouse and chicken limb buds. The middle line in the box plots represents the median (50$^{th}$ percentile), whereas the lower and upper hinges corresponds to the first (25$^{th}$ percentile) and third (75$^{th}$ percentile) quartiles, respectively. The upper whisker extends from the hinge to the largest value within 1.5 × IQR, while the lower whisker extends from the hinge to the smallest value within 1.5xIQR of the hinge. All data

points with values outside the whiskers are outliers and plotted individually. The minima and maxima of the box plots are determined after excluding the outliers. Mouse forelimb bud at E9.75, E10.5, and E11.5 show the best correspondence with chicken wing buds at HH20, HH22, and late-HH24, respectively. **c** Heatmap shows the expression pattern of highly conserved *Hoxa* (left panel) and *Hoxd* (right panel) gene clusters in mouse and chick limb buds. The scale represents min-max transformed values of transcript levels. The dynamic expression patterns of *Hoxa* and *Hoxd* genes reveals the high conservation of colinear expression in both species, which corroborates the developmental correspondence between the two species. **d** Line plots show the *z* normalized temporal expression profiles of key genes in the SHH and BMP pathway that are part of the self-regulatory signaling system, further strengthening the molecular correspondence of the orthologous mouse and chicken limb bud stages. Source data are provided as a Source Data file.

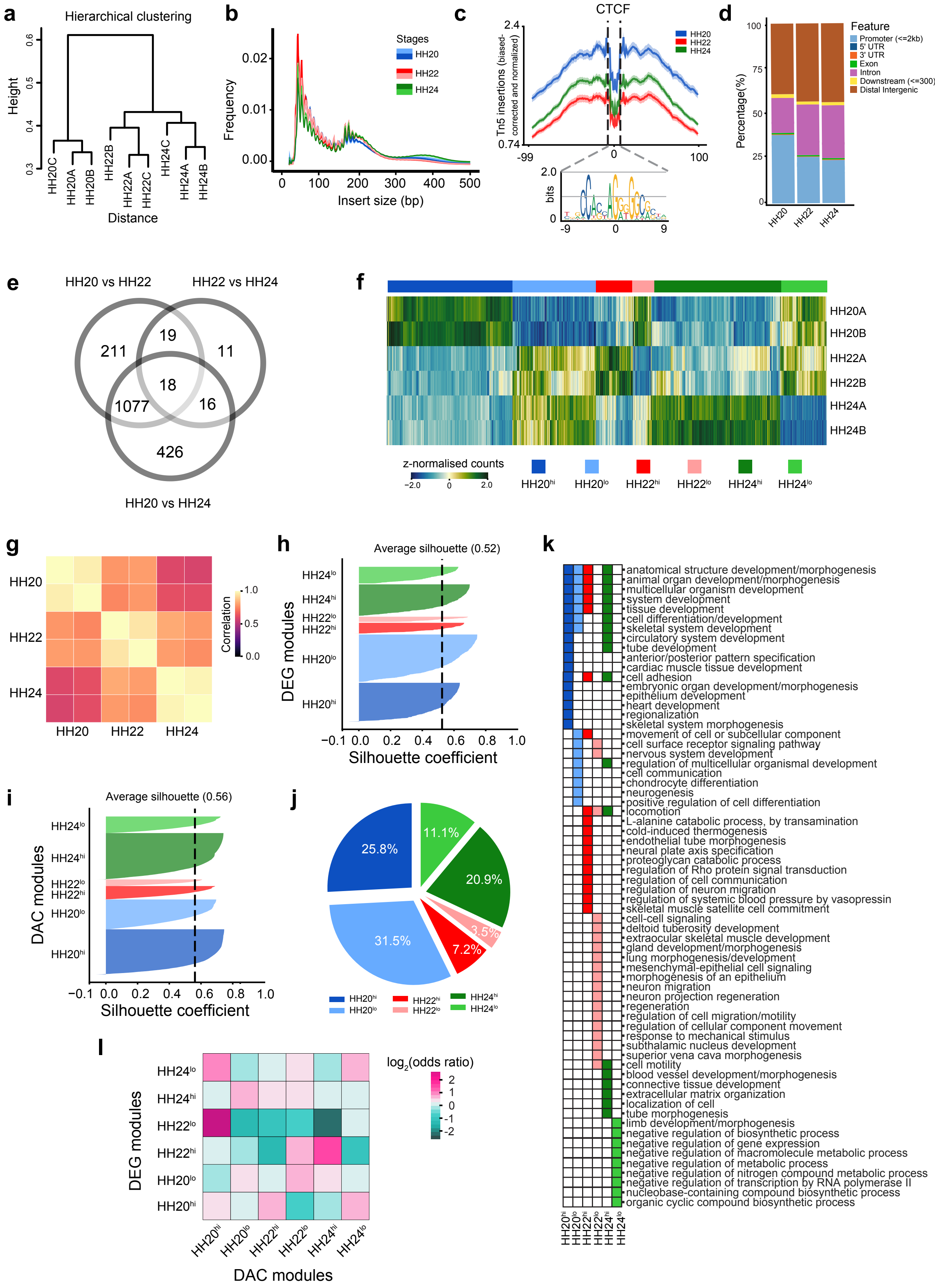
a
Hierarchical clustering
Height
Distance
HH20C
HH20A
HH20B
HH22B
HH22A
HH22C
HH24C
HH24A
HH24B
b
Stages
HH20
HH22
HH24
Frequency
Insert size (bp)
c
CTCF
Tn5 insertions (biased-corrected and normalized)
bits
d
Feature
Promoter (<=2kb)
5' UTR
3' UTR
Exon
Intron
Downstream (<=300)
Distal Intergenic
Percentage(%)
e
HH20 vs HH22
HH22 vs HH24
HH20 vs HH24
211
19
11
18
1077
16
426
f
HH20A
HH20B
HH22A
HH22B
HH24A
HH24B
z-normalised counts
g
Correlation
h
Average silhouette (0.52)
DEG modules
Silhouette coefficient
i
Average silhouette (0.56)
DAC modules
Silhouette coefficient
j
25.8%
11.1%
20.9%
3.5%
7.2%
31.5%
k
anatomical structure development/morphogenesis
animal organ development/morphogenesis
multicellular organism development
system development
tissue development
cell differentiation/development
skeletal system development
circulatory system development
tube development
anterior/posterior pattern specification
cardiac muscle tissue development
cell adhesion
embryonic organ development/morphogenesis
epithelium development
heart development
regionalization
skeletal system morphogenesis
movement of cell or subcellular component
cell surface receptor signaling pathway
nervous system development
regulation of multicellular organismal development
cell communication
chondrocyte differentiation
neurogenesis
positive regulation of cell differentiation
locomotion
L-alanine catabolic process, by transamination
cold-induced thermogenesis
endothelial tube morphogenesis
neural plate axis specification
proteoglycan catabolic process
regulation of Rho protein signal transduction
regulation of cell communication
regulation of neuron migration
regulation of systemic blood pressure by vasopressin
skeletal muscle satellite cell commitment
cell-cell signaling
deltoid tuberosity development
extraocular skeletal muscle development
gland development/morphogenesis
lung morphogenesis/development
mesenchymal-epithelial cell signaling
morphogenesis of an epithelium
neuron migration
neuron projection regeneration
regeneration
regulation of cell migration/motility
regulation of cellular component movement
response to mechanical stimulus
subthalamic nucleus development
superior vena cava morphogenesis
cell motility
blood vessel development/morphogenesis
connective tissue development
extracellular matrix organization
localization of cell
tube morphogenesis
limb development/morphogenesis
negative regulation of biosynthetic process
negative regulation of gene expression
negative regulation of macromolecule metabolic process
negative regulation of metabolic process
negative regulation of nitrogen compound metabolic process
negative regulation of transcription by RNA polymerase II
nucleobase-containing compound biosynthetic process
organic cyclic compound biosynthetic process
l
log2(odds ratio)
DEG modules
DAC modules

**Supplementary Figure 4. Analysis of the gene expression and chromatin remodelling dynamics during chicken wing bud development. a** Dendrogram represents the hierarchical clustering of the RNA-seq replicates (n=3) revealing low intra-stage variation. **b** Insert size frequency of ATAC-seq fragments showing nucleosome periodicity with mono- and di-nucleosomal fragments. **c** Aggregate ATAC-seq footprint centred on accessible sites around TFBS of CTCF. Shown below is the consensus binding site for the CTCF complex. **d** Distribution of accessible chromatin sites into genomic regions in chicken wing buds at all three stages. **e** Three-way Venn diagram illustrates the overlap of differentially expressed genes (DEGs) across different pairwise comparisons. **f** Heatmap shows the relative accessibility of DACs during wing bud development. The DACs with similar temporal accessibility patterns were assigned to six different DAC modules. **g** Spearman's correlation coefficient (ρ) computed using normalized accessibility scores of DACs for all pairwise combinations of developmental stages. The correlation values range from 0 to 1 (scale bar). The replicates (n=2) per stage are highly correlated (>0.9) corroborating reproducibility. **h-i** Silhouette plot showing the silhouette coefficient of the DEG (panel h) and DAC modules (i). An average silhouette score of +0.56 and +0.52 for DAC and DEG modules highlights that the samples are far from the decision boundary between two neighbouring modules. **j** Pie chart shows the fraction of DEGs assigned to the different DEG modules. **k** Graph showing the top enriched GO terms for each of the six DEG modules. **l** Log-odds ratios for two-tailed Fisher's exact test of enrichment between pairs of DAC and DEG modules. These correspond to the p-values shown in Fig. 3f, where an adjusted p-value <0.05 is considered significant. DAC: differentially

accessible chromatin regions, DEG: differentially expressed genes. Source data are provided as a Source Data file.

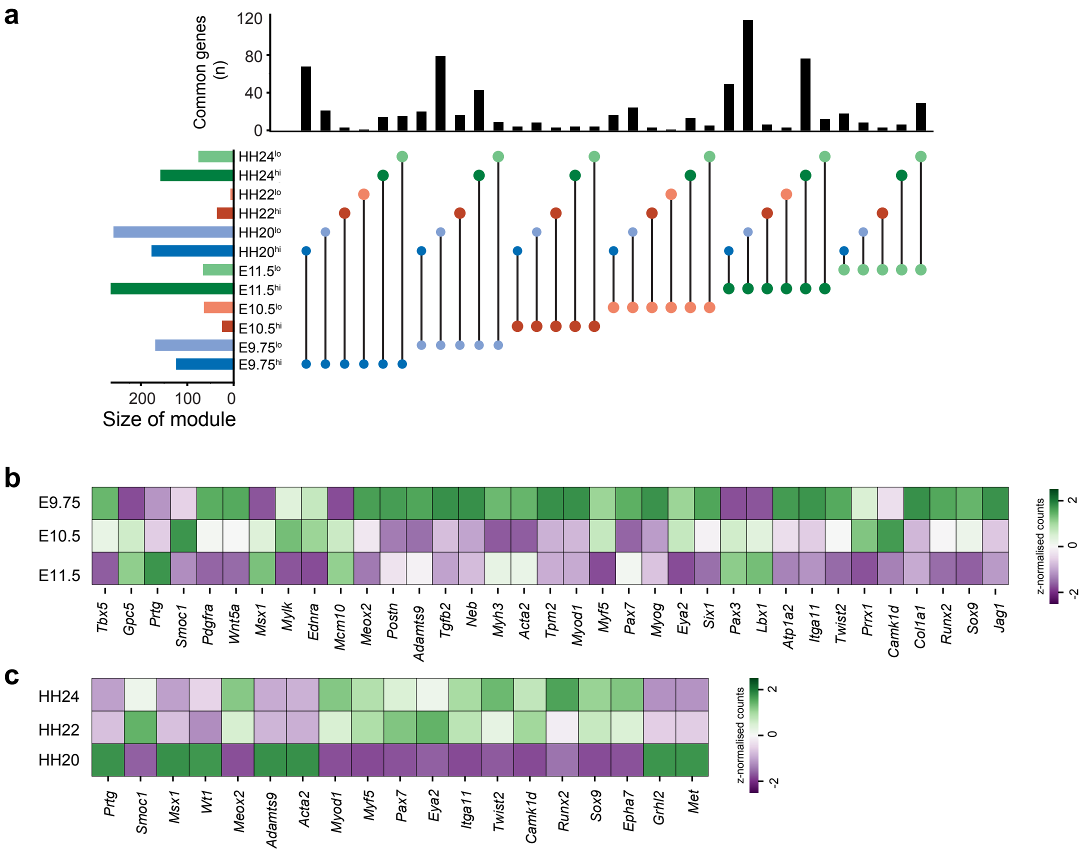


**Supplementary Figure 5. Variation in the temporal dynamics of gene expression among orthologous genes of mouse and chicken. a** Intersection of orthologous DEGs of the mm-gg category within the DEG modules of mouse and chicken limb buds. Vertical black bars represent the number of DEGs shared between two modules as indicated schematically by the coloured dots connected by a vertical line. The colour of dots is in accordance with the colour of DEG modules. Horizontal bars represent the size of the individual module. **b** Heatmap showing the *z* normalized expression for DEGs encoding limb key regulator genes with significant temporal changes in gene expression during mouse forelimb bud development. **c** Heatmap showing the *z* normalized expression for DEGs encoding limb key regulator genes

with significant temporal changes in gene expression during chicken wing bud development. DEG: differentially expressed genes. Source data are provided as a Source Data file.

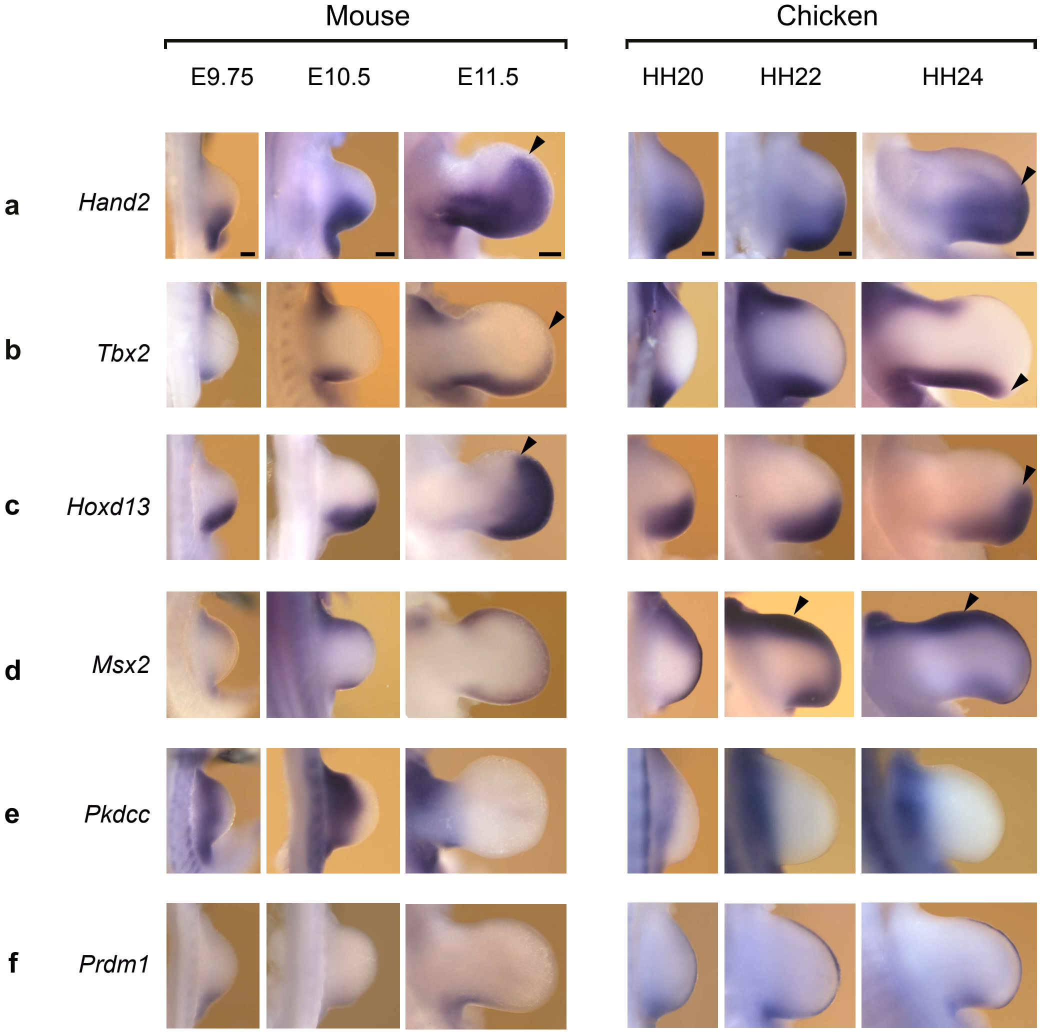


**Supplementary Figure 6. Comparative analysis of the spatial expression of developmental genes in mouse forelimb and chicken wing buds.** Whole-mount *in situ* hybridization showing spatio-temporal expression at developmentally equivalent stages of mouse forelimb (left column: E9.75, E10.5, E11.5) and chicken wing buds (right column: HH20, HH22, late-HH24). The key genes and their corresponding/closest DEG modules are **a** *Hand2* (n=3, E9.75$^{lo}$/HH24$^{hi}$), **b** *Tbx2* (n=3, E9.75$^{lo}$/HH20$^{hi}$), **c** *Hoxd13* (n=3, E11.5$^{hi}$/HH20$^{lo}$), **d** *Msx2* (n=3, E9.75$^{lo}$/HH20$^{hi}$), **e** *Pkdcc* (n=3, E11.5$^{lo}$/HH24$^{hi}$) and **f** *Prdm1* (n=3, E9.75$^{hi}$/HH24$^{lo}$). Arrowheads indicate

differences in anterior expression boundaries within the developing autopod of both species (panels a-c) and the anteriorly expanded expression domain in chicken wing buds (panel d). DEG: differentially expressed genes, Scale bars: 250μm.

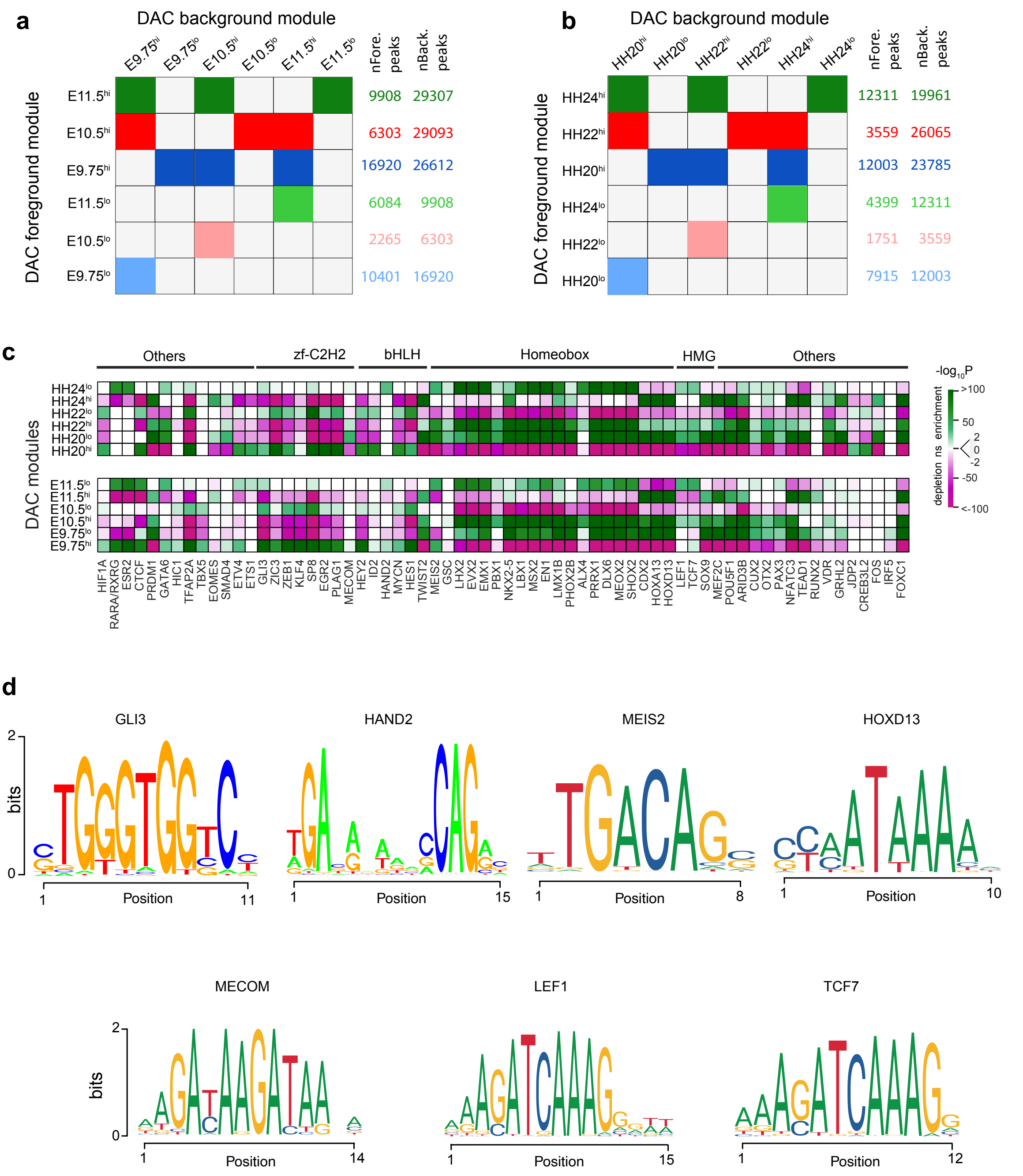


**Supplementary Figure 7. Computational prediction of TF binding sites provides evidence for *cis*-regulatory importance of DAC modules a-b.** Background set selection for TFBS enrichment analysis (Fig. 5) in mouse (panel a) and chicken (panel b) DAC modules. For each foreground DAC module (rows), the background DAC modules used (columns) are indicated as coloured boxes. The nFore.peaks and

nBack.peaks. represent the number of peaks in the foreground and background modules. These sets of foreground and background peaks were used to compute enrichment and depletion of TFBS within DAC modules as shown in Fig. 5a and Supplementary Fig. 7c. **c** Heatmap showing the significant TFBS enrichment (green) and depletion (magenta) for the six DAC modules in chicken (upper panel) and mouse limb buds (lower panel). The enrichment patterns for specific TFs are overall rather complementary between hi and lo DAC modules at the same stage. **d** Representative motif LOGOS showing information content of the consensus sequence for specific TF binding site motifs (related to Fig. 5b, c). DAC: differentially accessible chromatin regions. Source data are provided as a Source Data file.

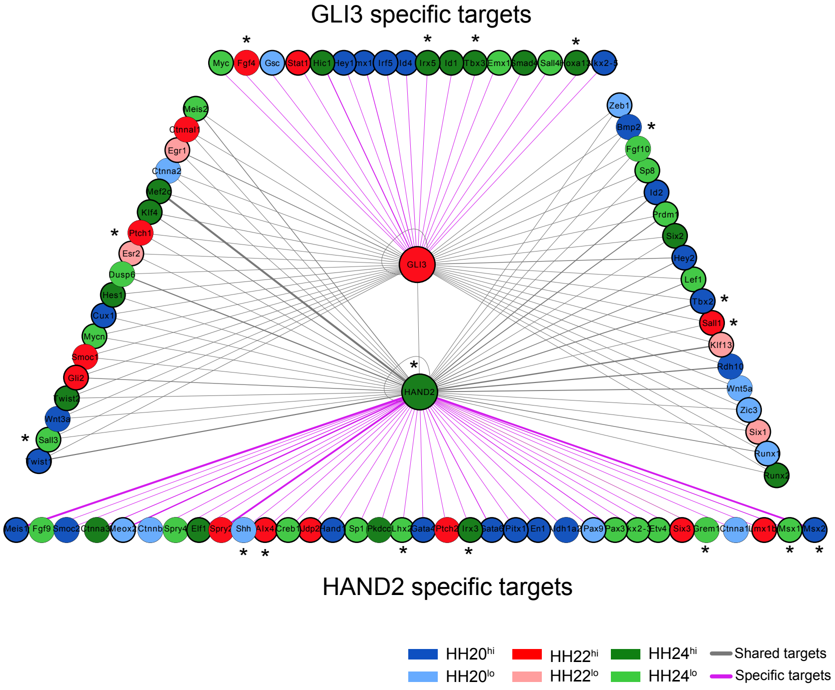


**Supplementary Figure 8. *In silico* identification of candidate GLI3 and HAND2 target genes in chicken wing buds.** Identification of *cis*-regulatory interactions of the GLI3 and HAND2 transcriptional regulators with a select set of genes expressed in limb buds (Supplementary Table 6). Circles represent nodes while the interactions are represented by solid lines (edges). GLI3 and HAND2 are source nodes and their candidate target genes are genes linked to those accessible regions with GLI3 and/or HAND2 TF binding motifs. The edges of the network represent motifs and are weighted (thickness of line) based on the number of TF binding motifs within the accessible regions linked to a target gene, thereby supporting a connection. GLI3 and HAND2 specific interactions are shown by magenta-coloured lines while shared interactions are represented by grey lines. The colour code of the candidate target genes corresponds to the closest DEG module (see Fig. 3b,c) based on the correlation

of their gene expression with DEG cluster centroids. The target genes encoding transcriptional regulators are outlined in bold. Target genes that have been experimentally validated in mouse limb buds are indicated by asterisks. DEG: differentially expressed genes. Source data are provided as a Source Data file.

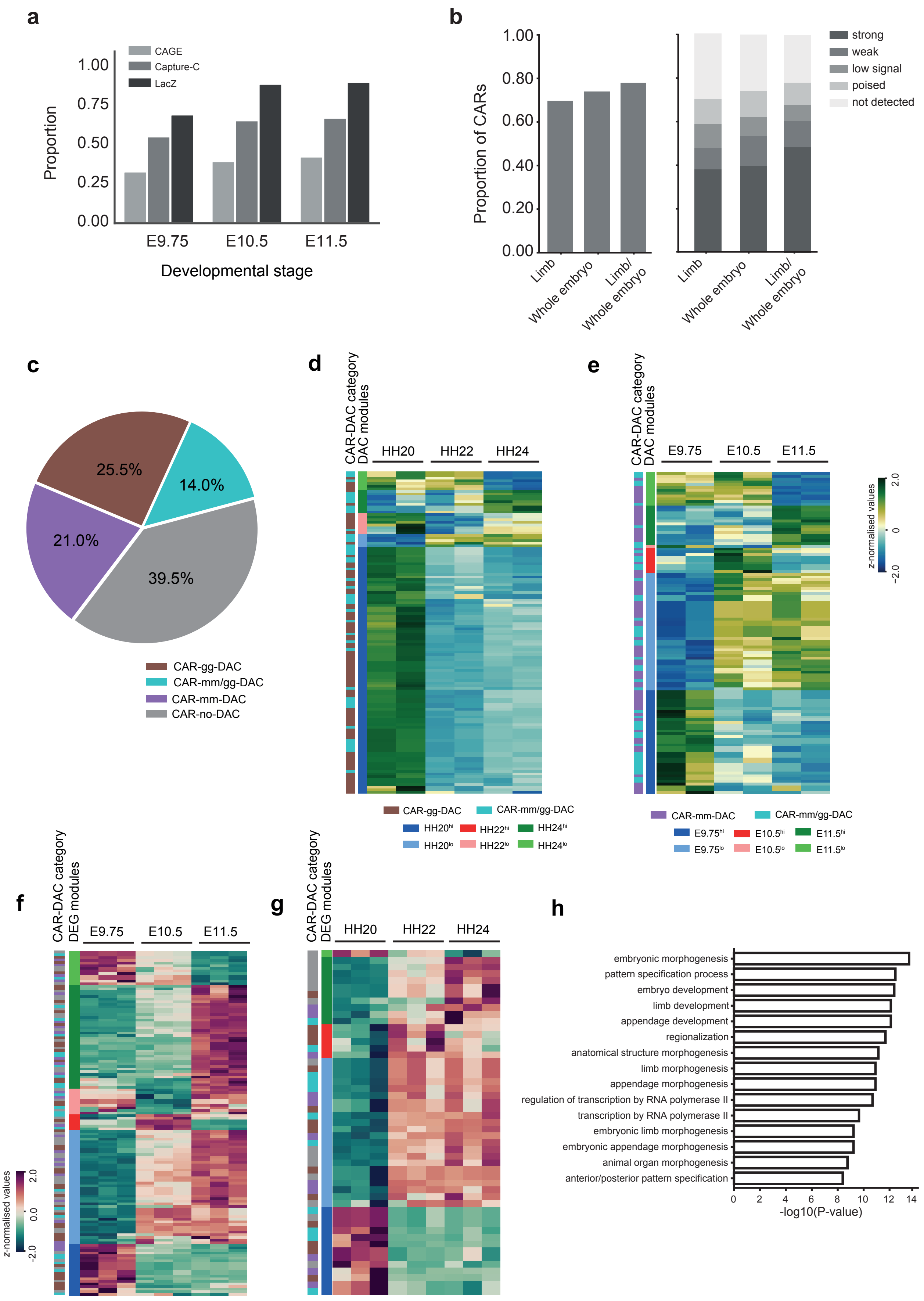
a
CAGE
Capture-C
LacZ
Proportion
1.00
0.75
0.50
0.25
0.00
E9.75
E10.5
E11.5
Developmental stage
b
Proportion of CARs
1.00
0.80
0.60
0.40
0.20
0.00
Limb
Whole embryo
Limb/ Whole embryo
strong
weak
low signal
poised
not detected
c
25.5%
14.0%
21.0%
39.5%
CAR-gg-DAC
CAR-mm/gg-DAC
CAR-mm-DAC
CAR-no-DAC
d
CAR-DAC category
DAC modules
HH20
HH22
HH24
CAR-gg-DAC
CAR-mm/gg-DAC
HH20hi
HH22hi
HH24hi
HH20lo
HH22lo
HH24lo
e
CAR-DAC category
DAC modules
E9.75
E10.5
E11.5
z-normalised values
2.0
0
−2.0
CAR-mm-DAC
CAR-mm/gg-DAC
E9.75hi
E10.5hi
E11.5hi
E9.75lo
E10.5lo
E11.5lo
f
CAR-DAC category
DEG modules
E9.75
E10.5
E11.5
z-normalised values
2.0
0.0
-2.0
g
CAR-DAC category
DEG modules
HH20
HH22
HH24
h
embryonic morphogenesis
pattern specification process
embryo development
limb development
appendage development
regionalization
anatomical structure morphogenesis
limb morphogenesis
appendage morphogenesis
regulation of transcription by RNA polymerase II
transcription by RNA polymerase II
embryonic limb morphogenesis
embryonic appendage morphogenesis
animal organ morphogenesis
anterior/posterior pattern specification
0
2
4
6
8
10
12
14
-log10(P-value)

**Supplementary Figure 9. Characterization of the chicken accelerated regions.**

**a** Bar plot showing the proportion of the assembled set of previously identified mouse limb bud enhancers (*lacZ*, Capture-C, and CAGE) accessible across the three mouse forelimb bud stages. **b** Bar plot (left panel) showing the proportion of CARs overlapping with published putative enhancers predicted in chicken embryo and/or limb buds using combinatorial patterns of epigenomic signatures (see results for details). Right panel: stacked bar plot illustrating proportion of CARs in different enhancer activity states, with strong enhancers being over-represented (Supplementary Table 2). **c** Pie chart shows the fraction of CARs in different CAR-DAC categories. **d** Heatmap shows the relative accessibility of CAR-associated DACs across chicken wing bud development. **e** Heatmap shows the relative accessibility of DACs across mouse forelimb development that associate to mouse orthologues of CARs. The vertical bars show the annotations of the DACs with respect to the mouse DAC modules (right bar) and CAR-DAC categories (left bar). **f-g** Heatmaps illustrating the relative gene expression trajectories across developmental stages for CAR-associated DEGs in developing mouse forelimb (panel f) and chicken wing buds (panel g). The vertical bars show the annotations of the DEGs with respect to the DEG modules (right bar) and CAR-DAC categories (left bar). **h** Top-enriched GO terms corresponding to CAR-associated DEGs. CAR: chicken accelerated region; DAC: differentially accessible chromatin regions, DEG: differentially expressed genes, GO: Gene Ontology. Source data are provided as a Source Data file.

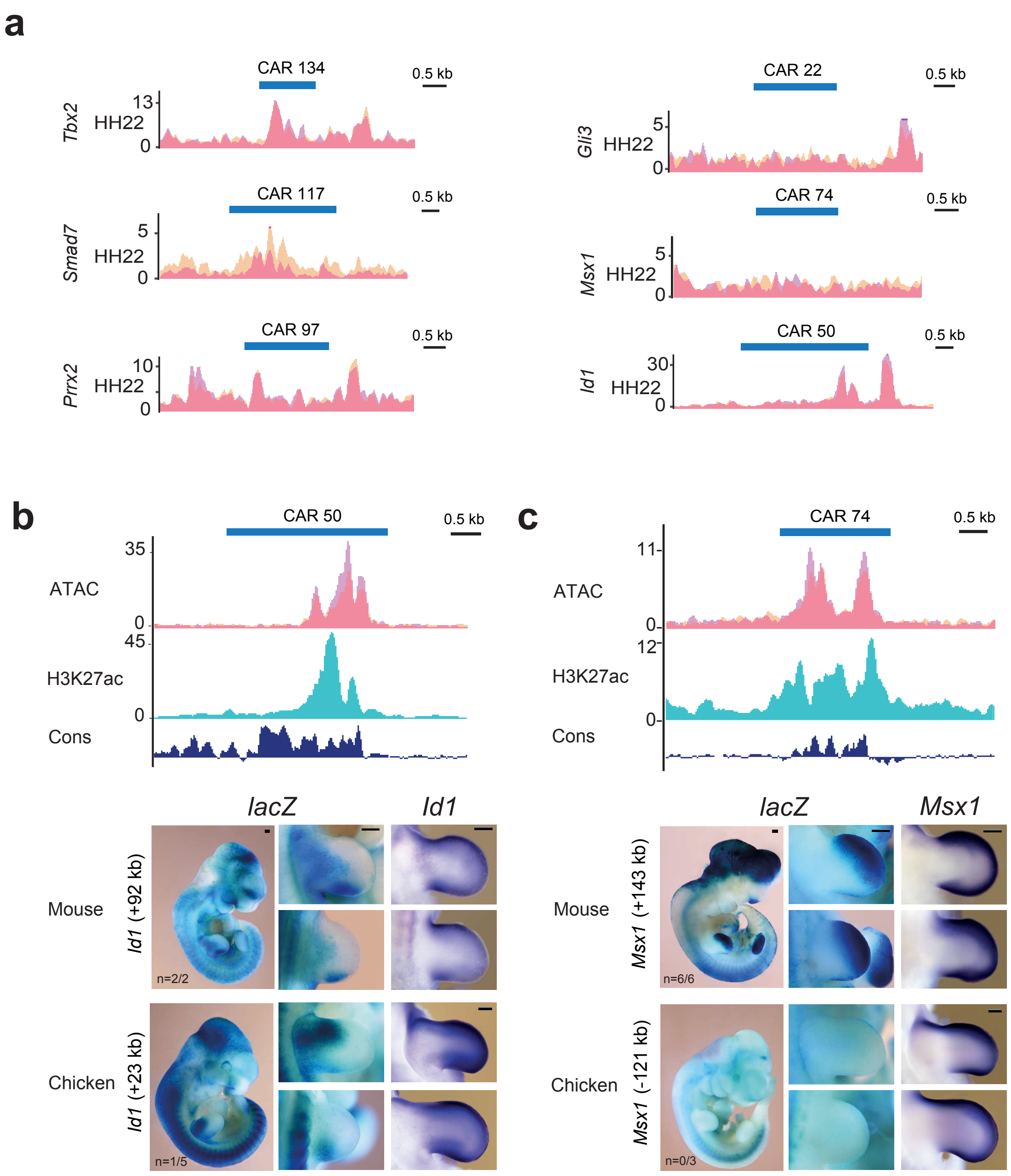


**Supplementary Figure 10. Divergent enhancer activity of CARs and their mouse orthologues.** **a** ATAC-seq profiles of the genomic regions overlapping CARs in chicken wing buds (HH22, related to Fig. 7). ATAC-seq signals from replicates are represented with two distinct shades, namely peach (RGB: 246, 202, 160) and magenta (RGB: 246, 160, 243). While the regions overlaying accessibility signals from

both replicates appear in dark pink (RGB: 235, 127, 153). **b-c** CARs lack enhancer activity (*Id1*: n=1/5, *Msx1*: n=0/3) in contrast to their mouse orthologues (*Id1*: n=2/2, *Msx1*: n=6/6). ATAC-seq (magenta) and histone H3K27ac signals (cyan) reveal that the orthologous mouse enhancers are part of active chromatin in forelimb buds at E10.5. Blue bars indicate the regions orthologous to the CARs. *lacZ* panels: the mouse enhancer and the chicken CAR activities were determined using transgenic mouse *lacZ* reporter assays. CAR50 and its mouse orthologue are linked to the *Id1* gene (panel **b**). CAR74 and its mouse orthologue are linked to the *Msx1* gene (panel **c**). *In situ* panels: spatial expression of the associated gene in mouse and chicken fore/wing and hindlimb buds at orthologous stages (E11.5/HH24). CAR: Chicken Accelerated Region, scale bars: 250 µm.

**Supplementary Table 1:** Probes generated as part of this study for whole-mount RNA *in situ* hybridization.

| Gene | mRNA ID | Forward primer | Reverse primer | Product size | Species |
|---|---|---|---|---|---|
| *Prdm1* | XM_003641038 | TATGAAAATGGACA TGGAGG | TAGGATTTCTTT CACACTGT | 655 | Chicken |
| *Pkdcc* | XM_040666455 | GCTCTATGGCTAT TGTTACC | CCTGTCCAAGT CGTCTGGTT | 759 | Chicken |
| *Msx1* | NM_205488 | GCCAGAAGCAGTA CCTGTCC | AATGGCCACAG GTTAACAGC | 572 | Chicken |
| *Prrx2* | NM_001293098 | AAGGTAGAGTCAA GTCCCAGTCCG | GTT TTC TGC TCA TCT CGC AAC | 773 | Chicken |
| *Prrx2* | XM_006497806 | AAA GAG TTC AGC CTA CAC CAC AG | GTG AGT TCC T TG GCG GAC TC | 302 | Mouse |

**Supplementary Table 2:** Over-representation tests for putative enhancers of different categories in CARs (chicken accelerated regions) using GAT, which estimates statistical significance based on simulation and controls for multiple tests using the false discovery rate (q-value <0.05; related to Supplementary Fig. 9b).

| Enhancer categories | Observed length (bp) | Expected length (bp) | p-value | q-value (FDR) | Fold |
|---|---|---|---|---|---|
| WholeEmbryo_E1 | 52665 | 26287 | 0.0010 | 0.0010 | 2.0034 |
| WholeEmbryo_E2 | 74842 | 70023 | 0.3300 | 0.3300 | 1.0688 |
| WholeEmbryo_E3 | 133990 | 160319 | 0.0860 | 0.0860 | 0.8358 |
| WholeEmbryo_E4 | 20336 | 12823 | 0.0930 | 0.0930 | 1.5858 |
| Limb_E1 | 44490 | 23162 | 0.0010 | 0.0010 | 1.9208 |
| Limb_E2 | 50333 | 53733 | 0.3760 | 0.3760 | 0.9367 |
| Limb_E3 | 134591 | 156148 | 0.1380 | 0.1380 | 0.8619 |
| Limb_E4 | 19348 | 14563 | 0.1890 | 0.1890 | 1.3285 |
| Limb_WholeEmbryo_E1 | 64264 | 35976 | 0.0010 | 0.0010 | 1.7863 |
| Limb_WholeEmbryo_E2 | 90570 | 89517 | 0.4730 | 0.4730 | 1.0118 |
| Limb_WholeEmbryo_E3 | 143791 | 177505 | 0.0470 | 0.0470 | 0.8101 |
| Limb_WholeEmbryo_E4 | 24937 | 19053 | 0.1580 | 0.1580 | 1.3088 |

**Supplementary Table 3:** Over-representation tests for putative enhancers of different categories in chicken DACs using GAT, which estimates statistical significance based on simulation and make adjustments for multiple tests using the false discovery rate (q-value<0.05).

| **Enhancer categories** | **Observed length (bp)** | **Expected length (bp)** | **p-value** | **q-value (FDR)** | **Fold** |
|---|---|---|---|---|---|
| WholeEmbryo_E1 | 9505353 | 2491097 | 0.00100 | 0.001000 | 3.8157 |
| WholeEmbryo_E2 | 7566677 | 6939012 | 0.00100 | 0.001000 | 1.0905 |
| WholeEmbryo_E3 | 10091304 | 16452314 | 0.00100 | 0.001000 | 0.6134 |
| WholeEmbryo_E4 | 2397877 | 1181629 | 0.00100 | 0.001000 | 2.0293 |
| Limb_E1 | 11639933 | 2235625 | 0.00100 | 0.001000 | 5.2066 |
| Limb_E2 | 6986416 | 5188787 | 0.00100 | 0.001000 | 1.3464 |
| Limb_E3 | 8078272 | 16179999 | 0.00100 | 0.001000 | 0.4993 |
| Limb_E4 | 2452223 | 1329196 | 0.00100 | 0.001000 | 1.8449 |
| Limb_WholeEmbryo_E1 | 13324045 | 3436506 | 0.00100 | 0.001000 | 3.8772 |
| Limb_WholeEmbryo_E2 | 10695860 | 8758458 | 0.00100 | 0.001000 | 1.2212 |
| Limb_WholeEmbryo_E3 | 11219187 | 18063756 | 0.00100 | 0.001000 | 0.6211 |
| Limb_WholeEmbryo_E4 | 3088534 | 1762362 | 0.00100 | 0.001000 | 1.7525 |

**Supplementary Table 4:** *lacZ* reporter constructs to assay enhancer activities in transgenic mouse embryos (Fig. 7 and Supplementary Fig. 10). The reverse and forward primers for cloning the orthologous genomic regions and the genomic coordinates of the putative enhancers (mm10 and galGal5) are indicated.

| CAR IDs | Target gene | *G. gallus* Forward (5'-3') | *G. gallus* Reverse (5'-3') | Genomic Coordinates (galGal5) | Size (bp) | *M. musculus* Forward (5'-3') | *M. musculus* Reverse (5'-3') | Genomic Coordinates (mm10) | Size (bp) |
|---|---|---|---|---|---|---|---|---|---|
| CAR74 | *msx1* | TGCTCCTTAGT TCGGCTGAG | AACAAGCAAG CAAACAAACA | chr4:79278224-79279921 | 1698 | GGAAGACTTG GGCACTCGTA | TCGCAAGAGGG GAATATCAC | chr5:37967097-37969393 | 2297 |
| CAR117 | *smad7* | TGGCTTTCTGA GGTTCAATG | TGGGGACAGG TATACCCAAG | chrZ:1195557-1198625 | 3069 | ACACTGCCGC TGAGGTTTAC | GAGACAAGCCC CACACAGAT | chr18:75206816-75210168 | 3353 |
| CAR22 | *gli3* | TGCATCTCATT AGCAAAGATGA A | GGTGCCCTTG AATAGTCTTCC | chr2:51113590-51115563 | 1974 | GGCTTTCTGG GAGTTTAGGG | TAGGGCAAGCT GCTCTATCC | chr13:15342429-15344998 | 2570 |
| CAR50 | *id1* | TGTAACAGCCA GGGAGAAGG | TCCACGGCCA TCGATTC | chr20:10230886-10234419 | 3534 | GTTAAGTGACA GGGCCAGGA | GGGCTCACAAC CAAAAGAAA | chr2:152828602-152832428 | 3827 |
| CAR97 | *prrx2* | GTCCTCACCAC ATCCCATTT | TTTATTGGCCC CAATGTTTG | chr17:6117578-6119474 | 1897 | TTTCCTCTCAG GGTCCTTCA | CTCTGAGCCAC CTCTCACCT | chr2:30833198-30839295 | 6098 |
| CAR134 | *tbx2* | ATTGACCTATC GGGGTTTCC | CAGAAGGAAA AGTTAATCGAG CTT | chr19:7804918-7806088 | 1171 | CCCCTCCACTC TGATGACTC | GTCAGGACCAG GTCAGGATG | chr11:85630872-85633430 | 2559 |
