# Supplementary material for: Conserved and species-specific chromatin remodeling and regulatory dynamics during mouse and chicken limb bud development
Source: Nat Commun. 2021 Sep 28;12:5685. doi: 10.1038/s41467-021-25935-3 (PMC8479071; doi:10.1038/s41467-021-25935-3)
Supplement: Supplementary file 3 — Description of Additional Supplementary Files [file 41467_2021_25935_MOESM3_ESM.pdf]

Description of Additional Supplementary Files

File Name: Supplementary Data 1
Description: List of differentially expressed genes (DEG) obtained from RNA-seq of developing mouse forelimb buds, related to Fig. 1. Chromosomal coordinates of the DEGs, DEG modules, and transcription factor (TF) family information is shown.

File Name: Supplementary Data 2
Description: Gene ontology terms for DEG modules of the mouse, related to Fig. 1. To get significant terms (p-value ≤0.05), all the annotated genes of Mus musculus were used as background to calculate enrichment and the adjustments were made for multiple comparisons. GO accession, GO term, evidence code, p-value and genes associated with each term are indicated.

File Name: Supplementary Data 3
Description: Accessible regions as obtained by ATAC-seq during mouse forelimb bud development, related to Fig. 2. Peak ID, peak coordinates, differential accessible information, associated gene symbol, gene coordinates, DEG modules, TF family, and Entrez ID details are shown.

File Name: Supplementary Data 4
Description: Accessible regions as obtained by ATAC-seq during chicken wing bud development, related to Fig. 3. Peak ID, peak coordinates, differential accessible information, associated gene symbol, gene coordinates, DEG modules, TF family, and Entrez ID details are shown.

File Name: Supplementary Data 5
Description: List of genes of an orthologous set of DEGs (OSD), related to Fig. 4. All the genes of both, mm_only and gg_only are listed. The details of orthologous ids, their expression cluster, and TF family are indicated in both species. The genes involved in mediating "abnormal limb morphology" with MP:0002109 accession from Mouse Genome Informatics (MGI) are also specified.

File Name: Supplementary Data 6
Description: List of select genes used for HAND2 and GLI3 candidate target identification, related to Fig. 6. The function of the select genes is obtained from David (https://david.ncifcrf.gov/summary.jsp) within *Mus musculus*.

File Name: Supplementary Data 7
Description: CARs identified through our computational pipeline, related to Fig. 7. The candidate genomic regions were tested for accelerated nucleotide substitutions in the chicken genome using likelihood ratio test as implemented in the PhyloP function of package PHAST. The false discovery rate (FDR) was computed using the Benjamini-Hochberg (BH) method.

File Name: Supplementary Data 8
Description: Association of chicken accelerated regions (CARs) with a putative enhancers identified using a combinatorial pattern of epigenomic signatures in developing limb and whole embryo at chicken developmental stages; b avian-specific highly conserved elements (ASHCEs). The majority of the CARs are supported by putative chicken embryonic and/or limb enhancers, related to Supplementary Fig. 9b.

File Name: Supplementary Data 9

Description: Association of chicken DACs (differentially accessible chromatin) with a putative enhancers identified using the combinatorial pattern of epigenomic signatures in developing limb and whole embryo at chicken developmental stages; b Avian-specific highly conserved elements (ASHCEs). The majority of the DACs are supported by putative chicken embryonic and/or limb enhancers.

File Name: Supplementary Data 10
Description: Association of chicken accelerated regions (CARs) with DACs/DEGs, related to Supplementary Fig. S9c-g. Whether CARs and their cognate mouse orthologs are differentially accessible in mouse and chicken respectively, is indicated. Among putative gene targets as identified by GREAT, differentially expressed genes (DEGs) in either of the species are shown for each CAR.
