# Supplementary material for: Conserved and species-specific chromatin remodeling and regulatory dynamics during mouse and chicken limb bud development
Source: Nat Commun. 2021 Sep 28;12:5685. doi: 10.1038/s41467-021-25935-3 (PMC8479071; doi:10.1038/s41467-021-25935-3)
Supplement: Supplementary file 14 — Reporting Summary [file 41467_2021_25935_MOESM14_ESM.pdf]

nature research

Corresponding author(s): Shalu Jhanwar, Aimée Zuniga, Rolf Zeller

Last updated by author(s): Aug 10, 2021

Reporting Summary

Nature Research wishes to improve the reproducibility of the work that we publish. This form provides structure for consistency and transparency in reporting. For further information on Nature Research policies, see our Editorial Policies and the Editorial Policy Checklist.

Statistics

For all statistical analyses, confirm that the following items are present in the figure legend, table legend, main text, or Methods section.

n/a	Confirmed	
☐	☒	The exact sample size (*n*) for each experimental group/condition, given as a discrete number and unit of measurement
☐	☒	A statement on whether measurements were taken from distinct samples or whether the same sample was measured repeatedly
☐	☒	The statistical test(s) used AND whether they are one- or two-sided<br>*Only common tests should be described solely by name; describe more complex techniques in the Methods section.*
☒	☐	A description of all covariates tested
☐	☒	A description of any assumptions or corrections, such as tests of normality and adjustment for multiple comparisons
☐	☒	A full description of the statistical parameters including central tendency (e.g. means) or other basic estimates (e.g. regression coefficient) AND variation (e.g. standard deviation) or associated estimates of uncertainty (e.g. confidence intervals)
☐	☒	For null hypothesis testing, the test statistic (e.g. *F*, *t*, *r*) with confidence intervals, effect sizes, degrees of freedom and *P* value noted<br>*Give P values as exact values whenever suitable.*
☒	☐	For Bayesian analysis, information on the choice of priors and Markov chain Monte Carlo settings
☒	☐	For hierarchical and complex designs, identification of the appropriate level for tests and full reporting of outcomes
☐	☒	Estimates of effect sizes (e.g. Cohen's *d*, Pearson's *r*), indicating how they were calculated

Our web collection on statistics for biologists contains articles on many of the points above.

Software and code

Policy information about availability of computer code

Data collection	No software was used for data collection.
Data analysis	Software used for next generation sequencing data analysis and genome sequence analysis were FASTQC v0.11.4, Trim galore v0.6.2 , Bowtie2 v2.2.9, Picard v2.8.0, SAMtools, MACS2 v2.1.1, BEDTools v2.26.0, edgeR, STAR v2.5.2, RSEM v1.3.0, Analysis of Motif Enrichment (AME) v5.0.4, MEME v5.0.4, ENSEMBL (release 91), edgeR v3.22.0, Regulatory Genomics Toolbox, and PHAST. For experiments, primers were designed using web-based Interface Primer 3. Custom codes are available at Zenodo (https://doi.org/10.5281/zenodo.5172892).

For manuscripts utilizing custom algorithms or software that are central to the research but not yet described in published literature, software must be made available to editors and reviewers. We strongly encourage code deposition in a community repository (e.g. GitHub). See the Nature Research guidelines for submitting code & software for further information.

Data

Policy information about availability of data

All manuscripts must include a data availability statement. This statement should provide the following information, where applicable:

- Accession codes, unique identifiers, or web links for publicly available datasets
- A list of figures that have associated raw data
- A description of any restrictions on data availability

The newly sequenced genome-wide RNA-seq and ATAC-seq datasets of mouse and chicken limb buds generated as part of the study have been deposited to Gene Expression Omnibus (GEO) public repository. The RNA-seq datasets of mouse and chicken developmental stages (E9.75, E10.5, E11.5, HH20, HH22, HH24) can be accessed with the accession number GSE164737 (https://www.ncbi.nlm.nih.gov/geo/query/acc.cgi?acc=GSE164737) . Whereas, ATAC-seq datasets of corresponding stages of mouse and chicken can be accessed at GSE164736 (https://www.ncbi.nlm.nih.gov/geo/query/acc.cgi?acc=GSE164736). All the newly sequenced datasets are freely accessible without any restrictions. Source data are provided with this paper.

In addition the following publicly available databases were used: Mouse Genome Informatics database (www.informatics.jax.org/), ENCODE (https://www.encodeproject.org/data/), Animal TFDB v3.0 (http://bioinfo.life.hust.edu.cn/AnimalTFDB/), JASPAR database (http://jaspar.genereg.net).

Field-specific reporting

Please select the one below that is the best fit for your research. If you are not sure, read the appropriate sections before making your selection.

☒ Life sciences ☐ Behavioural & social sciences ☐ Ecological, evolutionary & environmental sciences

For a reference copy of the document with all sections, see nature.com/documents/nr-reporting-summary-flat.pdf

Life sciences study design

All studies must disclose on these points even when the disclosure is negative.

Sample size

For ATAC-seq, 75,000 cells were processed per developmental stage of mouse forelimb and chicken wing bud as described in Buenrostro et al., 2015. For each sample, two independent biological replicates (n=2) were generated that allowed to determine reproducible signal.
For transcriptome analysis, 500ng total RNA was used to prepare RNA-seq libraries using NEB non-directional NEBNext® Ultra™ II RNA Library Prep (E7775), using the polyA mRNA workflow and 9 cycles of PCR amplification. Three biological replicates (n=3) per developmental stage of mouse forelimb and chicken wing bud were generated to get robust outcomes as specified in the ENCODE guidelines (https://www.encodeproject.org/about/experiment-guidelines/).
For qualitative whole-mount in situ (WISH) analysis, n≥3 replicates per sample of mouse and chicken embryos were analyzed. Gene expression patterns in embryos are extremely robust and based on our previous experience and the standard in the field as little to no variability is observed between stage-matched embryos (Benazet et al. 2009 10.1126/science.1168755). It is also important to note that (WISH) provides spatial information, but is not quantitative with respect to gene expression levels. For lacZ reporter assays of enhancer activities, the standard sample size to determine tissue-specific enhancer activities is n≥3 transgenic founder embryos that express the lacZ reporter in the tissue of interest (e.g. the limb bud). For reference see Visel A, et al. 2007 10.1093/nar/gkl822.

Data exclusions

None

Replication

Normalized genome-wide ATAC-seq signal between biological replicates was compared using Spearman rank-order correlation coefficient and the replicates (n=2 biological replicates) were found to be consistent with one another. For RNA-seq (n=3 biological replicates), principal components analysis and hierarchical clustering of the gene expression profiles established consistency among biological replicates.
For WISH, the reproducibility of gene expression patterns was assessed by comparing biological replicates and performing minimally two independent experiments for mouse forelimb and chicken wing buds.
For lacZ reporter assays, each transgenic founder embryo is an independent replicate as each insertion in the genome is unique. Variation can be observed due to position effects of transgene insertion, therefore a limb bud enhancer activity was considered robust when the activity domain was reproduced in minimally three founder embryos. An enhancer was considered variable/divergent or weak/non-functional if either no consistent or no activity was observed in limb bud. The fraction of the embryos with limb expression is indicated as n=x/y with x indicating the number of embryos with limb bud lacZ activity and y indicating the total number of embryos with lacZ activity (including also non-limb bud tissues).

Randomization

This is a comparative developmental study. Therefore, no randomization was possible as the limb bud samples need to age-matched according to the orthologous mouse and chicken forelimb and wing bud stages for analysis.

Blinding

It is impossible to blind the analysis as without using age-matched orthologous stages of mouse and chicken limb buds the study could not be conducted. This applies to the ATAC-seq, RNA-seq and WISH analysis.
The lacZ reporter activities was done blinded as founder embryos were stained and expression patterns scored prior to determining the genotypes (transgenic versus non-transgenic embryos).

Reporting for specific materials, systems and methods

We require information from authors about some types of materials, experimental systems and methods used in many studies. Here, indicate whether each material, system or method listed is relevant to your study. If you are not sure if a list item applies to your research, read the appropriate section before selecting a response.

Materials & experimental systems

n/a	Involved in the study	
☒	☐	Antibodies
☒	☐	Eukaryotic cell lines
☒	☐	Palaeontology and archaeology
☐	☒	Animals and other organisms
☒	☐	Human research participants
☒	☐	Clinical data
☒	☐	Dual use research of concern

Methods

n/a	Involved in the study	
☐	☒	ChIP-seq
☒	☐	Flow cytometry
☒	☐	MRI-based neuroimaging

Animals and other organisms

Policy information about studies involving animals; ARRIVE guidelines recommended for reporting animal research

Laboratory animals	The RjOrl:SWISS mouse strain was used for all mouse experiments. Mouse embryos of both sexes were collected from timed matings at embryonic days E9.75 (28-30 somites), E10.5 (34-36 somites) and E11.5 (43-45 somites) and used for analysis. For chicken, fertilized White Leghorn eggs (Animalco, Switzerland) were incubated, embryos of both sexes were isolated at the Hamburger-Hamilton stages HH20, HH22, and HH24 and used for analysis. Forelimb and wing buds were used for RNA-seq, ATAC-seq and whole embryos for comparative whole mount in situ analysis.
Wild animals	No wild animals were used in the study.
Field-collected samples	No field collected samples were used in the study.
Ethics oversight	All animal experiments were performed in accordance with Swiss laws and approved by the regulatory and ethic committees/ authorities of the Regional Commission on Animal Experimentation and the Cantonal Veterinary Office of the city of Basel under mandate from the confederation (mouse and chicken embryos).

Note that full information on the approval of the study protocol must also be provided in the manuscript.

ChIP-seq

Data deposition

☒ Confirm that both raw and final processed data have been deposited in a public database such as GEO.

☒ Confirm that you have deposited or provided access to graph files (e.g. BED files) for the called peaks.

Data access links
May remain private before publication.

RNA-seq - GSE164737 (https://www.ncbi.nlm.nih.gov/geo/query/acc.cgi?acc=GSE164737)
ATAC-seq - GSE164736 (https://www.ncbi.nlm.nih.gov/geo/query/acc.cgi?acc=GSE164736)

Files in database submission

The RNA-seq files linked with GEO accession GSE164737 are:

Raw FASTQ files
HFK5FBGX5_E975A_sequence.txt.gz
HV2VFBGX3_E975A_sequence.txt.gz
HFK5FBGX5_E975B_sequence.txt.gz
HV2VFBGX3_E975B_sequence.txt.gz
HFK5FBGX5_E975C_sequence.txt.gz
HV2VFBGX3_E975C_sequence.txt.gz
HFK5FBGX5_E105A_sequence.txt.gz
HV2VFBGX3_E105A_sequence.txt.gz
HFK5FBGX5_E105B_sequence.txt.gz
HV2VFBGX3_E105B_sequence.txt.gz
HFK5FBGX5_E105C_sequence.txt.gz
HV2VFBGX3_E105C_sequence.txt.gz
HFK5FBGX5_E115A_sequence.txt.gz
HV2VFBGX3_E115A_sequence.txt.gz
HFK5FBGX5_E115B_sequence.txt.gz
HV2VFBGX3_E115B_sequence.txt.gz
HFK5FBGX5_E115C_sequence.txt.gz
HV2VFBGX3_E115C_sequence.txt.gz
HFK5FBGX5_HH20A_sequence.txt.gz
HV2VFBGX3_HH20A_sequence.txt.gz
HFK5FBGX5_HH20B_sequence.txt.gz
HV2VFBGX3_HH20B_sequence.txt.gz
HFK5FBGX5_HH20C_sequence.txt.gz
HV2VFBGX3_HH20C_sequence.txt.gz
HFK5FBGX5_HH22A_sequence.txt.gz
HV2VFBGX3_HH22A_sequence.txt.gz
HFK5FBGX5_HH22B_sequence.txt.gz
HV2VFBGX3_HH22B_sequence.txt.gz
HFK5FBGX5_HH22C_sequence.txt.gz
HV2VFBGX3_HH22C_sequence.txt.gz
HFK5FBGX5_HH24A_sequence.txt.gz
HV2VFBGX3_HH24A_sequence.txt.gz
HFK5FBGX5_HH24B_sequence.txt.gz
HV2VFBGX3_HH24B_sequence.txt.gz
HFK5FBGX5_HH24C_sequence.txt.gz
HV2VFBGX3_HH24C_sequence.txt.gz

Processed files
GSM5017903_E975AAligned_dpProfiles_RNA.bw
GSM5017904_E975BAligned_dpProfiles_RNA.bw

GSM5017905_E975CAligned_dpProfiles_RNA.bw
GSM5017906_E105AAligned_dpProfiles_RNA.bw
GSM5017907_E105BAligned_dpProfiles_RNA.bw
GSM5017908_E105CAligned_dpProfiles_RNA.bw
GSM5017909_E115AAligned_dpProfiles_RNA.bw
GSM5017910_E115BAligned_dpProfiles_RNA.bw
GSM5017911_E115CAligned_dpProfiles_RNA.bw
GSM5017912_HH1920AAligned_dpProfiles_RNA.bw
GSM5017913_HH1920BAligned_dpProfiles_RNA.bw
GSM5017914_HH1920CAligned_dpProfiles_RNA.bw
GSM5017915_HH22AAligned_dpProfiles_RNA.bw
GSM5017916_HH22BAligned_dpProfiles_RNA.bw
GSM5017917_HH22CAligned_dpProfiles_RNA.bw
GSM5017918_HH24AAligned_dpProfiles_RNA.bw
GSM5017919_HH24BAligned_dpProfiles_RNA.bw
GSM5017920_HH24CAligned_dpProfiles_RNA.bw

The ATAC-seq files linked with GEO accession GSE164736 are:

Raw files
e975_ATAC_rep1.R1.fastq.gz
e975_ATAC_rep1.R2.fastq.gz
e975_ATAC_rep2.R1.fastq.gz
e975_ATAC_rep2.R2.fastq.gz
e105_ATAC_rep1.R1.fastq.gz
e105_ATAC_rep1.R2.fastq.gz
e105_ATAC_rep2.R1.fastq.gz
e105_ATAC_rep2.R2.fastq.gz
e115_ATAC_rep1.R1.fastq.gz
e115_ATAC_rep1.R2.fastq.gz
e115_ATAC_rep2.R1.fastq.gz
e115_ATAC_rep2.R2.fastq.gz
h20_ATAC_rep1.R1.fastq.gz
h20_ATAC_rep1.R2.fastq.gz
h20_ATAC_rep2.R1.fastq.gz
h20_ATAC_rep2.R2.fastq.gz
h22_ATAC_rep1.R1.fastq.gz
h22_ATAC_rep1.R2.fastq.gz
h22_ATAC_rep2.R1.fastq.gz
h22_ATAC_rep2.R2.fastq.gz
h24_ATAC_rep1.R1.fastq.gz
h24_ATAC_rep1.R2.fastq.gz
h24_ATAC_rep2.R1.fastq.gz
h24_ATAC_rep2.R2.fastq.gz

Processed files
GSM5017891_e975_ATAC_rep1_dpProfiles.bw
GSM5017892_e975_ATAC_rep2_dpProfiles.bw
GSM5017893_e105_ATAC_rep1_dpProfiles.bw
GSM5017894_e105_ATAC_rep2_dpProfiles.bw
GSM5017895_e115_ATAC_rep1_dpProfiles.bw
GSM5017896_e115_ATAC_rep2_dpProfiles.bw
GSM5017897_h20_ATAC_rep1_dpProfiles.bw
GSM5017898_h20_ATAC_rep2_dpProfiles.bw
GSM5017899_h22_ATAC_rep1_dpProfiles.bw
GSM5017900_h22_ATAC_rep2_dpProfiles.bw
GSM5017901_h24_ATAC_rep1_dpProfiles.bw
GSM5017902_h24_ATAC_rep2_dpProfiles.bw

Genome browser session
(e.g. UCSC)

N/A

Methodology

Replicates

High quality sequencing data was generated in replicates for each stage sample of ATAC-seq (n=2) and RNA-seq (n=3). For ATAC-seq samples, normalized genome-wide ATAC-seq signal between biological replicates was compared using Spearman rank-order correlation coefficient and the replicates were found to be consistent with one another.
For RNA-seq samples, principal components analysis and hierarchical clustering of the gene expression profiles established consistency among biological replicates.

Sequencing depth

For ATAC-seq, raw paired-end reads of 41 bp for developing mouse forelimb bud (E9.75, E10.5 and E11.5) and chicken wing bud (HH20, HH22 and HH24) stages were profiled (>150 million reads per sample) and evaluated for quality using Trim galore v0.6.2 wrapper for Cutadapt (Martin, 2011) with --nextera --gzip --paired settings. High quality reads (>99%) were aligned using Bowtie v2.2.9 on reference genomes of mouse (mm10) and chicken (galGal5), yielding >70% of uniquely aligned reads per sample.
For RNA-seq, raw single-end sequencing reads of 85 bp for developing mouse forelimb bud (E9.75, E10.5 and E11.5) and chicken

wing bud (HH20, HH22 and HH24) stages were profiled and subjected to quality check using Trim Galore v 0.4.1. High-quality sequencing reads were aligned on reference genome and transcriptome of mouse and chicken using STAR v2.5.2, finally yielding >85% of uniquely aligned reads per sample.

Antibodies	No antibodies were used for RNA-seq and ATAC-seq.
Peak calling parameters	For each sample of ATAC-seq, enriched regions (peaks) of accessible chromatin were detected using MACS2 v2.1.1 with the --nomodel --shift -75 --extsize 150 -B --SPMR --keep-dup all --call-summits settings.
Data quality	Significantly enriched peaks were called using MACS2 v2.1.1 for each sample. Then high confident reproducible peaks between biological replicates was determined using Irreproducible Discovery Rate (Li et al. 2011) following ENCODE standards and only reproducible peaks were considered for downstream ATAC-seq module analysis.<br>For gene expression, principal components analysis and hierarchical clustering of the gene expression profiles established consistency among biological replicates. Differentially expressed genes (DEGs) were identified using a GLM framework with edgeR. Significant DEGs were required to demonstrate a linear absolute FC cutoff of ≥ 1.5 and adjusted p-value ≤0.05.
Software	The quality of the raw paired-end sequencing reads in both species was determined by FASTQC v0.11.4. Nextera primer sequences were removed with the Trim galore v0.6.2 wrapper tool for Cutadapt using the --nextera --gzip --paired settings. Subsequent read alignments and post-processing was performed following ENCODE data processing standards (https://www.encodeproject.org/data-standards/). Briefly, trimmed reads were aligned with either the GRCm38/mm10 (mouse) or Gallus_gallus-5.0/galGal5 (chicken) genome build using Bowtie2 v2.2.9 with the -t -p 4 -X 2000 --mm -q --phred33-quals settings. Removal of PCR duplicates and generation of fragment size statistics were performed using the Picard v2.8.0. SAMtools command utilities were used to remove mitochondrial and low-quality reads. Enriched regions (peaks) of accessible chromatin were detected using MACS2 v2.1.1 with the --nomodel --shift -75 --extsize 150 -B --SPMR --keep-dup all --call-summits settings. Consistency between biological replicates was checked by Irreproducible Discovery Rate (IDR) and only reproducible peaks were considered for downstream analysis. Annotation of peaks in genomic regions was done using the R package ChIPseeker v1.18.0. Normalized genome-wide profiles of ATAC-seq were generated using DeepTools. For gene expression, high-quality sequencing reads were aligned to either mouse (mm10) or chicken (galGal5) reference genomes using STAR v2.5.2 aligner with --twopassMode Basic and --quantMode TranscriptomeSAM settings. After alignment, transcripts and gene-wise counts were computed using rsem-calculate-expression utility of RSEM v1.3.0. Raw counts were normalized using TMM and DEGs were identified using a GLM framework with edgeR. Genome-wide normalized signal coverage tracks were generated using DeepTools.
